# The Impact of Lifestyle Intervention on Dietary Quality among Rural Women with Previous Gestational Diabetes Mellitus—A Randomized Controlled Study

**DOI:** 10.3390/nu13082642

**Published:** 2021-07-30

**Authors:** Mingshu Li, Qian Lin, Jingcheng Shi, Yue Xi, Caihong Xiang, Cuiting Yong, Jia Guo

**Affiliations:** 1 Department of Nutrition Science and Food Hygiene, Xiangya School of Public Health, Central South University, Changsha 410078, China; limingshu@csu.edu.cn (M.L.); linqian@csu.edu.cn (Q.L.); xiyue0404@csu.edu.cn (Y.X.); xch0622@csu.edu.cn (C.X.); yongcuiting@csu.edu.cn (C.Y.); 2 Department of Epidemiology and Health Statistics, Xiangya School of Public Health, Central South University, Changsha 410078, China; shijch@csu.edu.cn; 3 Xiangya School of Nursing, Central South University, Changsha 410013, China

**Keywords:** dietary quality, lifestyle intervention, women with previous gestational diabetes mellitus (GDM), rural areas, randomized controlled study

## Abstract

Healthy diet is essential to type 2 diabetes mellitus (T2DM) prevention for women with previous gestational diabetes mellitus (GDM). To evaluate the effect of a lifestyle intervention program on diet quality for rural women who were previously diagnosed with GDM, we conducted a randomized controlled study in two counties located in south-central China. A total of 404 eligible women were allocated into an intervention group and control group. Participants in the intervention group received 6-month lifestyle intervention including six group seminars and eight telephone consultations. Dietary data were collected at baseline and 18 months via a 24 h dietary recall, and dietary quality was measured by two indicators, Chinese Healthy Eating Score (CHEI) and Minimum Dietary Diversity for Women (MDD-W). Baseline CHEI scores (54.4 vs. 53.5, *p* = 0.305) and the proportions of participants who met MDD-W (73.8% vs. 74.5%, *p* = 0.904) were comparable between the two groups. The intervention group achieved a higher CHEI score (62.2 vs. 58.9, *p* = 0.001) and higher MDD-W proportion (90.6% vs. 81.2%, *p* = 0.023) at 18 months. Lifestyle intervention was associated with the change of CHEI (*p* = 0.049) but not with MDD-W (*p* = 0.212). In conclusion, compared with usual care, lifestyle intervention resulted in greater improvement of dietary quality among rural women with previous GDM.

## 1. Introduction

Gestational diabetes mellitus (GDM) is a category of diabetes that is initially diagnosed in the second or third trimester of pregnancy [1,2]. It is an independent risk factor for many postpartum diseases, especially type 2 diabetes mellitus (T2DM) [3,4]. The 10-year T2DM cumulative incidence after delivery is 20% to 40% for women with GDM, and the incidence further increases to 58% when prolonged to 50 years [5,6].

Diet is critical to T2DM development [7,8,9]. A high-quality diet, featuring adequacy in food diversity and balance in foods/nutrients, is associated with a decreased risk of T2DM among people at high risk for T2DM [10,11]. Studies of lifestyle intervention have been implemented to prevent T2DM via improving diet (alone or with physical activity) [12,13,14]. For women with previous GDM, one of the most influential studies is the Diabetes Prevention Program Outcome Study, in which intensive lifestyle intervention induced a 35% reduction in T2DM risk during a 10-year follow-up period [15].

Previous studies suggested unsatisfactory diet behavior among women who were previously diagnosed as GDM, because they faced many obstacles to a healthy lifestyle: for example, fragmented medical care, time, and energy stress [16,17,18,19]. For women with previous GDM in rural areas of China, this issue is even more urgent. Swift social restructuring has brought an urbanized lifestyle (e.g., high-fat food) to rural residents, which in turn, results in a higher prevalence of chronic diseases including T2DM among them [1,20,21]. Meanwhile, inequal health resources and a low level of health literacy are significant barriers to a healthy lifestyle in rural areas [22,23]. Our previous study has demonstrated unsatisfactory diet quality among women with previous GDM in rural regions [24]. Currently, there is no report relevant to lifestyle intervention for this special population in China. Therefore, we conducted a randomized controlled trial to probe a proper lifestyle intervention pattern for women with previous GDM in rural areas and examine its impact on diet quality, physical activity, and physiological health outcomes. This paper aims to analyze the effect of lifestyle intervention on diet quality.

## 2. Materials and Methods

### 2.1. Study Design

A randomized controlled study was conducted in Youxian county and Yongding county; both counties are located in south-central China. Data collection and intervention implementation were conducted in General Hospital of Youxian County and Maternal and Children’s Hospital of Yongding District between September 2017 and January 2020. This study was approved by the ethical committee of Xiangya Nursing School of Central South University (No. 2016034). Detailed information of study protocol, including participants, lifestyle intervention, and primary and secondary outcomes, was described previously [25].

### 2.2. Participants

Women were enrolled from Youxian county and Yongding county. Those who met the following criteria were introduced to this study by trained nurses: (a) adult women with a history of GDM [1]; (b) at least six weeks after delivery; (c) intending to live in rural areas for at least 18 months; and (d) having access to telephone. Those who met any one of the following criteria were excluded from this study: (a) currently pregnant or plan to conceive in the next 18 months; (b) diagnosed with diabetes before pregnancy or after delivery; and (c) physical or cognitive disability.

### 2.3. Randomization

Women were cluster-randomized into intervention group or control group. There are 14 towns in Youxian county and 17 towns in Yongding county. A biostatistician (Wiley. J) assigned 8 towns in each county to the intervention group via Internet randomization protocol (http://stattrek.com/statistics/random-number-generator.aspx (accessed on 10 April 2017)). Women in other towns were allocated to the control group. Study was open to participants and professionals who conducted intervention, while blinded to data assessors.

### 2.4. Lifestyle Intervention

The 6-month lifestyle intervention program was designed according to theory of planned behavior (TBP) [26], which has been widely applied to change health-related behavior [27,28,29]. Women and their family members were invited to join 6 group seminars (at most 20 people per group, 90 min per session) for the first 10 weeks. In the first orientation seminar, the trainer introduced the relationship of GDM and postpartum T2DM and demonstrated the benefit of healthy lifestyle to participants. In the second seminar, the eating pattern recommended by national dietary guideline was introduced to the participants [30], and they were encouraged to analyze the barriers to healthy diet and set goals for dietary adjustment. The third session covered physical activities plan and barriers. The fourth session focused on stress assessment and management. The fifth seminar focused on family support. During this session, family members were encouraged to express their attitude and suggestions concerning participant’s lifestyle behavior. The barriers and solutions to healthier family meal/exercise were also discussed. In the last session, the strategies to maintain healthy lifestyle were introduced. Besides group seminars, 8 telephone consultations were also provided to review the progress and assist goal achievement. Five bi-weekly calls were conducted during first 3 months, and three monthly calls were conducted in the following 3 months.

### 2.5. Control Group

Participants in the control group received usual care, without group seminars or telephone consultations.

### 2.6. Data Collection

Sociodemographic information was collected at baseline. Dietary information, physical activity, and anthropometric parameters, including weight and body mass index (BMI), were gathered at 0 and 18 months after baseline.

#### 2.6.1. Dietary Intake

Dietary intake was collected via a 24 h dietary recall administered by trained investigators. Participants were required to take photos of all the foods and drinks they consumed in three consecutive days. They recalled the foods and their quantities with the visual reminder of the photos during the face-to-face interview. The nutrients from foods were estimated by NutriStar software (Shanghai Zhending Inc., Shanghai, China), which calculates energy and nutrients according to 2018 Chinese Food Composition Table [31]. Average daily intake of energy and food groups was used to assess dietary quality.

#### 2.6.2. Dietary Quality

Two indicators, the Chinese Healthy Eating Index (CHEI) and Minimum Dietary Diversity for Women (MDD-W), were applied to evaluate dietary quality [32,33].

CHEI was developed to measure the compliance to the Balanced Dietary Pattern advocated by Chinese Dietary Guidelines [30]. It has a continuous scoring system, in which 100 implies full adherence to dietary guidelines and optimal diet quality. There are 12 adequacy components and 5 moderation components in the index. Adequacy components include total grains, whole grains and mixed beans, tubers, total vegetables, dark vegetables, fruits, dairy, soybeans, fish and seafood, seeds and nuts, poultry, and eggs. Moderation components are red meat, cooking oils, sodium, added sugars, and alcohol. Scoring of each component was based on its quantity (standard portion/1000 kal). A maximum point of 5 or 10 was assigned where the intake met the recommendation for each component. Zero points were allotted for no intake (adequacy component) or severe excess (moderation component) (Supplementary Table S1). Points between 0 and maximum were scored proportionately. Total CHEI score was the sum of 17 components’ scores. 

MDD-W is a dichotomous indicator aiming to measure the adequacy of micronutrients for women aged 15 to 49. There are 10 predefined food groups: (1) grains, white roots and tubers, and plantains; (2) pulses; (3) nuts and seeds; (4) dairy; (5) meat, poultry, and fish; (6) eggs; (7) dark green leafy vegetables; (8) other vitamin A-rich fruits and vegetables; (9) other vegetables; and (10) other fruits. A cutoff of >15 g was used to define an amount enough to count towards food group diversity. Scores of 1 were assigned if women consumed at least five of the ten food groups. Otherwise, 0 points were allotted.

#### 2.6.3. Anthropometric Parameters

To measure weight and height, participants were required to dress lightly and be barefooted. Weight was examined by Tanita BC-718 (Tanita, Tokyo, Japan) and recorded to the nearest 0.1 kg. Height was measured by a calibrated scale and was recorded to the nearest centimeter. BMI was calculated by dividing the weight by height (in meters squared) and was categorized into four levels: obese (≥28.0 kg/m^2^), overweight (24.0–27.9 kg/m^2^), normal (18.5–23.9 kg/m^2^), and underweight (≤18.5 kg/m^2^) [34]. 

#### 2.6.4. Physical Activity 

The International Physical Activity Questionnaire Short Form (IPAQ-SF) was implemented to collect the data of physical activity [35]. The time spent on vigorous activity, moderate activity, walking, and sitting over a week was reported by participants. Then, the metabolic equivalent of the task was calculated, and categories of physical activity (i.e., vigorous, moderate, low) were determined for each participant. 

#### 2.6.5. Sociodemographic Information

Sociodemographic data (e.g., age, ethnicity, education, occupation, civil status, family income, parity) were collected via a self-report questionnaire.

### 2.7. Outcomes

The primary outcome was the difference in CHEI score and proportion of participants reaching MDD-W after 18 months between the two groups. The secondary outcomes were the difference in energy and nutrient (i.e., carbohydrate, protein, fat, calcium, iron, zinc, vitamin A, thiamine, riboflavin, vitamin C, niacin, folate) intake between the two groups after 18 months.

### 2.8. Sample Size

The sample size was estimated based on previous report of CHEI score among Chinese adults who participated in China Health and Nutrition Survey (CHNS-2011), in which the mean CHEI score of rural people was 49.3 (9.5) [32]. A total sample size of 400 was required, assuming a 3-point increase after intervention and 20% drop-out rate, with Type 1 error 0.5 and power of the test 0.8 (GPower 3.1.9.2).

### 2.9. Statistical Analysis

Baseline continuous variates were described by mean (SD) or median (95% confidence interval). Categorial data were reported as proportions. CHEI score of the intervention group and the control group was compared by independent samples t-test. Participants were deemed to reach MDD-W if five (or more than five) food groups were consumed. The proportion of participants reaching the MDD-W was compared by chi-squared test. To explore the effect of lifestyle intervention to CHEI, a generalized linear mixed model (GLMM) was applied. Group, age, BMI, visit time, ethnicity, education level, occupational status, family monthly income, and parity were included as fixed effects. Binary logistical regression model was used to examine the contribution of above-mentioned factors to MDD-W. The energy and nutrient intake were classified into three categories, i.e., insufficient, adequate, and excessive. Insufficient intake was defined as less than 90% of estimated energy requirement (EER) or recommend nutrients intake (RNI) or less than the lower cutoff of AMDR [26]. Excessive intake was defined as more than 110% of EER/RNI or the higher cutoff of AMDR. The proportion of women who consumed adequate energy/nutrient was compared by chi-squared test. Statistical analysis was conducted by SPSS (version 24), and *p* value < 0.05 was considered statistically significant.

## 3. Results

### 3.1. Participants

**Figure 1:**
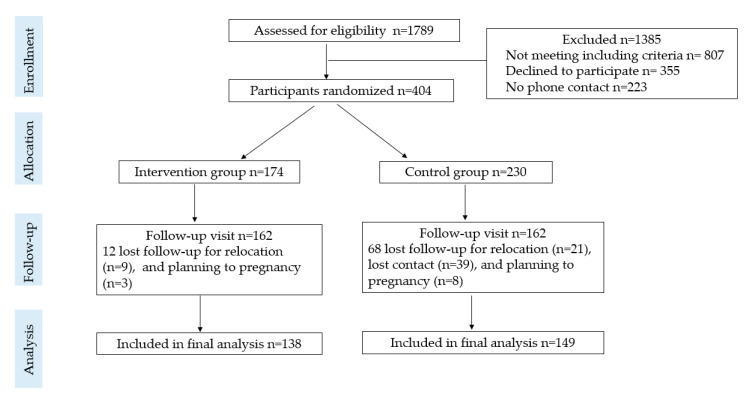
Flowchart of participant recruitment and allocation.

A total of 404 women were randomized in our study, and 324 (80.2%) of them completed follow-up for 18 months (Figure 1). We further excluded 37 participants because of incomplete dietary information; thus, 287 (intervention group *n* = 138, control group *n* = 149) were included in the final analysis.

The average age of the participants was 31.8 (range 19–44). The mean duration from GDM diagnosis to study enrollment was 1.9 years. Nearly half (48.1%) of the participants were of ethnic minorities. Most of them (78.0%) had an education level of senior high school or above (>9 years) (China has been implementing nine years of compulsory education since 1986). Two thirds of participants’ family monthly income surpassed CNY 3000 (equal to USD 460), which was the intermediate level of rural family income (per capita monthly income of rural households in Hunan province in 2017 was CNY 1077) [36]. Half of the women were of normal BMI, 31.0%, and 12.5% were overweight and obese (the prevalence of overweight and obesity among rural women in China was 49.8% and 15.6%, respectively, in 2018) [37]. Baseline characteristics were generally comparable between the two groups (Table 1). 

### 3.2. CHEI

The total CHEI score was low in both intervention and control groups (54.4 vs. 53.5, *p* = 0.305) at baseline. Two groups scored similarly on 17 components except for tubers (1.8 vs. 1.2, *p* = 0.008) and soybeans (2.4 vs. 1.8, *p* = 0.012). After 18 months, the intervention group achieved a higher CHEI score (62.2 vs. 58.9, *p* = 0.001). Among the components, the intervention group scored higher on total vegetables, soybeans, and red meat (*p* = 0.024, 0.015, 0.025) (Table 2). 

### 3.3. MDD-W

At baseline, the proportion of participants reaching the MDD-W was 73.8% and 74.5% in the intervention group and the control group (*p* = 0.904). A similar proportion of participants consumed 10 food groups except nuts and seeds (26.2% vs. 14.5% *p* = 0.016) and other vegetables (85.4% vs. 93.1%, *p* = 0.037). After 18 months, the proportion of participants reaching the MDD-W was 90.6% and 81.2% in the intervention group and the control group, with a significant difference (*p* = 0.023). A larger proportion of women in the intervention group consumed pulses at the follow-up visit (33.3% vs. 19.5%, *p* = 0.008) (Table 3). 

### 3.4. Association of CHEI, MDD-W with Lifestyle Intervention 

In GLMM, lifestyle intervention was found to influence total CHEI score significantly (*p* = 0.049) after other factors were included in the analysis, by increasing 1.5 points compared to non-intervention. Other prominent factors included visit time, age, BMI, and ethnicity. Lifestyle intervention was not associated with MDD-W status (0 or 1) in the logistical regression model (*p* = 0.212); the only significant factor for MDD-W was ethnicity (*p* = 0.027). 

### 3.5. Energy and Nutrient Intake

Overall baseline energy and nutrient intake were unsatisfactory (Supplementary Table S2). Nearly 80% of participants consumed excessive fat. Micronutrients were consumed inadequately. More than half of the women took calcium, vitamin A, thiamine, riboflavin, vitamin C, and folate insufficiently from food. There was no significant difference in energy, macronutrient, and micronutrient intake between the intervention group and the control group.

After 18 months, there was a significant decrease in the consumption of carbohydrates and fat (*p* = 0.000, 0.000) in the intervention group. Additionally, in this group, the quantity of micronutrients generally increased, while the changes were not significant. Compared with the control group, the intervention group consumed a significantly lower amount of carbohydrates (223.5 vs. 239.6, *p* = 0.038) (Supplementary Table S3). The proportion of participants who consumed adequate energy/nutrient at the follow-up visit between the two groups was similar (Supplementary Table S4).

## 4. Discussion

Our study demonstrated the impact of a 6-month lifestyle intervention on diet quality in women with a history of GDM. Both CHEI and MDD-W have improved more significantly in the intervention group than in the control group. Previous randomized controlled studies which applied lifestyle intervention for women with previous GDM examined the dietary change mainly via individual nutrient/food (e.g., carbohydrate, fat, vegetable) [38,39,40]. In MAGDA study, the Australian Recommended Food Score (ARFS) was used to measure comprehensive dietary quality [41]. In that study, a 6-month lifestyle intervention did not achieve significant changes in total ARFS at month 12, while that study only enrolled women within 12 months postpartum. We noticed the dietary behavior improved moderately in the control group. There were two possible reasons for this phenomenon. One was the Hawthorne effect: women regulated their lifestyle spontaneously after they were invited into this study [42]. The other reason was that the participants in the control group might have misreported their diet by exaggerating or omitting some foods (e.g., vegetables, red meat).

The dietary improvement observed in our study is potentially beneficial to T2DM prevention. Although the relationship between CHEI and MDD-W and T2DM risk is not conclusive, a recent cross-sectional study demonstrated that CHEI score was negatively related to metabolic syndrome [43,44]. Moreover, numerous studies suggested that eating behavior change is associated with glycemic improvement and lower T2DM incidence for women with or without GDM history [12,13,14,15,38,39,40]. From the perspective of food groups, the major foods that contributed to dietary quality improvement in our study were vegetables, red meat, and soybean. These three food groups have been confirmed to have a significant effect on T2DM [8,45,46,47]. For example, a daily consumption of 300 g of vegetable induces 9% lower risk of T2DM [8].

In our study, CHEI and MDD-W were applied to measure dietary quality from two different perspectives. CHEI reflects the adherence to the optimal dietary structure for the Chinese Balanced Dietary Pattern (BDP). The BDP is advocated by the Chinese Dietary Guideline and is based on the evidence from a national dietary survey and key health issues of the Chinese population [48]. A key characteristic of BDP is food variety. Specifically, five categories of food are encouraged to be consumed on a daily basis, i.e., grains, vegetables and fruits, animal food, soybeans and nuts, and cooking oil and salt [30]. Those five categories are embodied in CHEI with 17 food groups. To balance nutrient intake, food groups are classified into adequacy and moderation components. The baseline CHEI score is generally low among women in our study and is quite similar to the score in Chinese adults who participated in CHNS-2011 (mean value 54.6 vs. 52.4) [32]. MDD-W is used to examine the adequacy of micronutrients, considering that an insufficiency in major micronutrients among Chinese residents is prevalent. According to a national health survey, less than half of Chinese residents met the vitamin A, vitamin C, thiamine, riboflavin, and calcium intake recommendation [49]. Moreover, a decreasing number of Chinese adult females consumed adequate minerals and vitamins during the past decade [50]. Since the cutoff quantity for the MDD-W food groups count was the minimum, the baseline proportion of participants reaching MDD-W was already over 70%, which might partly explain why the lifestyle intervention was not associated with MDD-W in the logistical regression analysis.

After the intervention, the consumption of some food groups (e.g., vegetables, soybeans, and red meat) improved prominently, while whole grains and dairy were still consumed in poor quality. Grains are the traditional staple food for the Chinese, while mainly in refined form, e.g., white rice and noodles. According to the China Health and Nutrition Survey 1991–2011, the intake of whole grains among Chinese adults stayed at an extremely low level, 4 g/day in 1997 and 4.6 g/day in 2011 [51]. The low intake of whole grains is one of the most important risk factors related to the Chinese diabetes burden [51]. Another food consumed at an extremely low level is dairy. Nutrients in dairy-like whey proteins, vitamin D, and calcium are beneficial to insulin secretion and beta-cell function [52,53]. A daily intake of 400 to 600 g dairy would decrease the risk of T2DM progression by 6% [8]. Scarcity of supply was the major reason for low milk consumption in China decades ago, while currently, box-packed milk is available at nearly every grocery store/supermarket in the investigated towns at an affordable price [54]. Although participants in the intervention group have increased the intake of whole grains and milk substantially, the component food score was still low. To completely change whole grain and milk consumption habits, which have lasted for generations, we might need a longer intervention duration, as well as social/community support. 

According to the participants and their family members’ feedback at group seminars and during telephone consultations, the major barriers to a healthy diet among our participants included low disease risk perception, limited knowledge of healthy diet, food preferences of the family member, personal dietary habits, and time pressure. These barriers are consistent with previous qualitative studies [17,18,19]. Several elements may be required in a successful intervention scheme, e.g., exemplifying healthy meals, engaging family members, and improving perceived behavior control. In our study, the changes in micronutrients intake was not significant, which reflected a limitation of self-administered dietary modification. Thus, providing evidence-based daily menus might be crucial to further improve the dietary quality and clinical outcomes. Another element that might be considered in an interventional program in China is mobile social platform. Around 10% of participants discontinued our study since they had to work elsewhere. This is understandable given the high population mobility in China [55,56]. We suppose Wechat and other social platforms might help to improve retention rate in future studies. 

Our study has several limitations. Since the follow-up period of this study was 18 months, the baseline and follow-up data were collected in different seasons. Thus, food consumption change might be affected due to seasonal dietary habits: for example, seeds and nuts. Secondly, the dietary behavior of the control group might be influenced by the Hawthorne effect; thus, the difference of effect between the two groups could be underestimated. Moreover, the data accuracy might be undermined by memory lapses and incorrect quantification in the 24 h dietary recall, although we collected the dietary information with the help of an interviewer and photo images [57,58]. Fourthly, this study was conducted within a restricted region and restricted population; whether it could be generalized to other regions or people without GDM needs further study. Lastly, this study focuses on diet quality; the physiological health outcome is not reported here, and it would need to be reported in other studies.

## 5. Conclusions

This randomized controlled study demonstrated that for women with previous GDM in rural areas of China, a 6-month lifestyle intervention achieved greater diet quality improvement compared with usual care, which was potentially beneficial to T2DM prevention. 

## Figures and Tables

**Table 1:** Baseline characteristics of participants.

Variables	Participants Randomized (*n* = 404)			Participants Included in Final Analysis (*n* = 287)		
	Intervention *n* = 174	Control *n* = 230	*p*	Intervention *n* = 138	Control *n* = 149	*p*
Age (years)	31.7 (5.1)	30.9 (5.1)	0.104	32.1 (5.1)	31.6 (5.0)	0.376
Ethnic (%)			0.105			0.980
Han ethnicity	49.7	57.8		51.8	51.7	
Other ethnicities	50.3	42.2		48.2	48.3	
Education (%)			0.530			0.712
senior high school or above (9–12 years)	78.7	76.1		79.0	77.2	
junior high school or below (≤9 years)	21.3	23.9		21.0	22.8	
Monthly family income ($) (%)			0.214			0.828
≤420	29.7	25.7		31.8	30.6	
>420	70.3	74.3		68.2	69.4	
Occupation (%)			0.938			0.745
Unemployed	34.3	34.0		33.6	435.5	
Employed	65.9	66.0		66.4	64.5	
BMI	24.0 (3.6)	23.9 (3.7)	0.756	23.7 (3.4)	24.1 (3.7)	0.424
BMI (%)			0.091			0.026
Underweight	3.5	5.3		2.9	5.5	
Normal	54.7	45.1		59.6	43.4	
Overweight	26.2	36.7		24.3	38.6	
Obesity	15.7	12.8		13.2	12.4	
Parity (%)			0.408			0.385
1	35.1	38.9		41.5	39.5	
2	62.1	58.1		51.0	51.3	
>2	2.8	3		7.5	9.2	

**Table 2:** Change of CHEI score in the intervention group and control group.

Components/Total CHEI	Intervention		Control		*p* (Intervention vs. Control at Follow-Up Visit)
	Baseline	Follow-Up	Baseline	Follow-Up	
total grains	4.2 (0.9)	4.5 (0.9)	4.4 (0.8)	4.7 (0.6) *	0.116
whole grains and mixed beans	0.3 (0.8)	0.9 (1.7)	0.2 (0.6)	0.7 (1.5) *	0.300
tubers	1.8 (2.0)	1.3 (1.9) *	1.2 (1.8)	1.0 (1.6)	0.157
total vegetables	2.5 (1.2)	3.3(1.2) *	2.6 (1.3)	2.9 (1.4) *	0.024
dark vegetables	2.0 (1.6)	2.7 (1.8) *	2.1 (1.6)	2.4 (1.7)	0.105
fruits	2.3 (2.9)	4.7 (3.9) *	2.7 (3.2)	4.5 (3.7) *	0.591
eggs	2.1 (1.9)	2.9 (2.1) *	2.3(2.0)	2.9 (2.0) *	0.802
soybeans	2.4 (2.0)	2.8 (2.2)	1.8 (1.9)	2.2 (2.1)	0.015
dairy	0.4 (1.1)	0.9 (1.6) *	0.6(1.3)	0.8 (1.4)	0.290
seeds and nuts	1.7 (2.3)	0.9 (1.9) *	1.3 (2.2)	0.8 (1.8) *	0.635
fish and seafood	2.0 (2.1)	2.7 (2.2) *	1.6 (1.9)	2.4 (2.2) *	0.273
poultry	1.5 (2.2)	2.4 (2.4) *	1.4 (2.0)	1.9 (2.4) *	0.081
red meat	3.7 (0.9)	4.3 (0.8) *	3.9 (0.8)	4.1 (0.8) *	0.025
added sugars	5.0 (0.0)	5.0 (0.0) **	4.9 (0.3)	5.0 (0.1)	0.337
cooking oils	9.4 (1.5)	9.1 (1.5)	9.4 (1.6)	9.1 (1.7)	0.997
alcohol	5.0 (0.0)	5.0 (0.0)	5.0 (0.0)	5.0 (0.0) **	0.300
sodium	8.4 (2.6)	8.7 (2.3)	8.4 (2.6)	8.6 (2.4)	0.557
Total CHEI score	54.4 (7.4)	62.2 (8.9) *	53.5(7.6)	58.9 (8.4) *	0.001

* *p* < 0.05 between baseline and follow-up within intervention or control group; ** *t* value was not available because SD from both groups was 0.

**Table 3:** The proportion of participants who consumed MDD-W food groups.

Proportion	Intervention		Control		*p* (Intervention vs. Control at Follow-Up Visit)
	Baseline	Follow-Up	Baseline	Follow-Up	
Grains, white roots, and tubers	99.2	99.3	99.3	100	0.298
Meat, poultry, and fish	55.4	99.3 *	62.1	96.6 *	0.120
Dairy	23.1	31.9	26.2	26.2	0.286
Pulses	23.1	33.3	19.3	19.5	0.008
Nuts and seeds	26.2	9.4 *	14.5	9.4	0.994
Dark green leafy vegetables	72.3	73.9	73.1	69.8	0.439
Other vitamin A-rich fruits and vegetables	30.8	43.5 *	35.2	40.9	0.663
Other vegetables	85.4	94.9 *	93.1	94.6	0.910
Other fruits	48.5	65.2 *	52.4	62.4	0.622
Eggs	61.5	69.6	64.1	71.1	0.770
At least 5 groups	73.8	90.6	74.5	81.2	0.023

* *p* < 0.05 between baseline and follow-up within intervention or control group.

## Data Availability

Not applicable.

## Supplementary Materials

The following are available online at https://www.mdpi.com/article/10.3390/nu13082642/s1, Table S1: CHEI scoring system. Table S2: Baseline energy/nutrient intake. Table S3: Change of energy/nutrient intake in the intervention group and control group. Table S4: The proportion of women who consumed adequate energy/nutrient at baseline and follow-up visit.
