# Supplementary material for: The Impact of Lifestyle Intervention on Dietary Quality among Rural Women with Previous Gestational Diabetes Mellitus—A Randomized Controlled Study
Source: Nutrients. 2021 Jul 30;13(8):2642. doi: 10.3390/nu13082642 (PMC8402030; doi:10.3390/nu13082642)
Supplement: Supplementary file 1 [file nutrients-13-02642-s001.zip › nutrients-1315727-SI.pdf]

Supplementary Table S1. CHEI scoring system

Component	Maximum points	Standard for maximum point	Standard for zero point
Adequacy			
Total grains	5	≥2.5SP/1000 kcal	No intake
Whole Grains and mixed beans	5	≥0.6SP/1000 kcal	No intake
Tubers	5	≥0.3SP/1000 kcal	No intake
Total vegetables	5	≥1.9SP/1000 kcal	No intake
Dark vegetables	5	≥0.9SP/1000 kcal	No intake
Fruits	10	≥1.1SP/1000 kcal	No intake
Dairy	5	≥0.5SP/1000 kcal	No intake
Soybeans	5	≥0.4SP/1000 kcal	No intake
Fish and Seafood	5	≥0.6SP/1000 kcal	No intake
Poultry	5	≥0.3SP/1000 kcal	No intake
Eggs	5	≥0.5SP/1000 kcal	No intake
Seeds and Nuts	5	≥0.4SP/1000 kcal	No intake
Moderation			
Added sugars	5	≤10% of energy	≥20% of energy
Sodium	10	≤1000 mg/1000 kcal	≥3608 mg/1000 kcal
Cooking oils	10	≤15.6 g/1000 kcal	≥32.6 g/1000 kcal
Red meat	5	≤0.4 SP/1000 kcal	≥3.5 SP/1000 kcal
Alcohol	5	≤15 g	≥40g

Supplementary Table S2. Baseline energy / nutrient intake

Dietary parameter	Mean (SD)/ Median (Range)	Prevalence (%)			CDG recommendation (RNI/EER, AMDR)
		Insufficient	Adequate	Excessive	
Energy (kcal)	2028.9 (541.0)	43.8	26.5	29.7	1800/2100/2400*
Carbohydrate (%E)	52.1 (26.9-76.5)	41.0	53.1	5.9	50~65
Protein (g)	65.1 (23.4)	26.7	17.5.	55.8	55
Fat (%E)	35.6 (18.3-61.3)	0.7	20.6	78.7	20-30
Calcium (mg)	373.6 (177.7)	96.9	0.7	2.4	800
Iron (mg)	20.4 (7.9)	43.9	23.9	32.3	20
Zinc (mg)	12.4 (4.1)	7.0	5.2	87.8	7.5
Vitamin A (μg RAE)	508.3 (465.0)	73.8	9.4	16.8	700
Vitamin E (mg)	24.4 (7.5)	1.4	3.9	94.7	14
Thiamine (mg)	0.7 (0.3)	90.9	4.9	4.2	1.2
Riboflavin (mg)	0.8 (0.3)	85.7	8.4	5.9	1.2
Vitamin C (mg)	84.5 (60.1)	63.3	11.9	24.8	100
Niacin (mg NE)	19.7(7.5)	7.7	10.1	82.2	12
Folate (μg DFE)	137.4(88.3)	98.0	0	2.0	400

CDG, Chinese Dietary Guideline-2016; RNI, recommend nutrients intake; EER, estimated energy requirement; AMDR, acceptable macronutrient distribution ranges.

* estimated energy requirement according to different categories of physical activity

Supplementary Table S3. Change of energy/nutrients intake in the intervention and control group

Energy/nutrient	Intervention		Control		*p* (intervention vs control at follow-up visit)
	Baseline	Follow-up	Baseline	Follow-up	
Energy (kcal)	2041.5(578.2)	2017.2(506.0)	2017.2(506.0)	2037.9(574.6)	0.093
Carbohydrate (g)	258.7 (77.9)	223.5(59.8)	263.7 (89.9)	239.6(70.8)	0.038
Protein (g)	67.6 (24.6)	64.1 (22.5)	62.9 (21.9)	65.0 (25.2)	0.752
Fat (g)	84.7 (32.6)	59.1 (21.3)	80.2(27.6)	60.8(23.0)	0.496
Calcium (mg)	369.3 (174.9)	402.7 (203.9)	377.4 (180.6)	384.8(203.2)	0.456
Iron (mg)	20.9 (7.7)	21.6 (10.9)	19.9 (8.2)	21.9 (9.2)	0.790
Zinc (mg)	12.6 (4.32)	11.9 (3.7)	12.1 (3.76)	12.6 (3.9)	0.126
Vitamin A (μg RAE)	526.3 (540.4)	670.6(1537.9)	491.7 (384.0)	537.0(436.1)	0.310
Vitamin E (mg)	24.9(7.9)	23.3(6.9)	23.9(7.1)	22.6（6.9）	0.440
Thiamine (mg)	0.7 (0.3)	0.7（0.3）	0.7 (0.2)	0.7（0.3）	0.495
Riboflavin (mg)	0.7 (0.3)	0.8（0.7）	0.7 (0.3)	0.8（0.3）	0.630
Vitamin C (mg)	88.6 (67.7)	92.7 (77.7)	84.5 (52.3)	91.1 (91.3)	0.870
Niacin (mg NE)	20.263 (7.9)	18.9 (7.3)	19.11(7.0)	20.1 (6.6)	0.147
Folate (μg DFE)	143.179 (103.2)	169.3 (125.2)	131.207 (69.2)	162.3(118.3)	0.627

Supplementary Table S4. The proportion of the women who consumed adequate energy/nutrient at baseline and follow-up visit

Proportion	Intervention		Control		*p* (intervention vs control at follow-up visit)
	Baseline	Follow-up	Baseline	Follow-up	
Energy	23.5	31.4*	29.3	28.6	0.259
Carbohydrate (%E)	50.4	35.7*	55.7	36.9*	0.835
Protein	17.6	29.0	17.4	22.8	0.451
Fat (%E)	17.5	68.5*	23.4	68.8*	0.423
Calcium	0.7	2.2	0.7	4.7	0.465
Iron	21.3	23.9	26.2	24.2*	0.366
Zinc	3.6	3.6	6.7	5.4	0.715
Vitamin A	9.5	10.1	9.4	9.4	0.959
Vitamin E	3.7	2.2	4.0	6.7	0.182
Thiamine	5.8	7.2	4.0	9.4	0.656
Riboflavin	9.5	10.1	7.4	14.1	0.585
Vitamin C	15.3	8.0	8.7	14.8	0.122
Niacin	8.8	8.0	11.4	6.7	0.871
Folate	0	1.4	0	1.3	0.578

*$p<0.05$ between baseline and follow-up within intervention or control group
